# Multi-modality human phenotyping to examine subjective and objective health afflictions in former professional American-style football players: The In-Person Assessment (IPA) protocol

**DOI:** 10.1371/journal.pone.0265737

**Published:** 2022-03-31

**Authors:** Bryan Cortez, Chelsea Valdivia, Dylan Keating, Dean Marengi, Trevor Bates, Cheyenne Brown, Inana Dairi, Michael Doyle, Robyn Keske, Ann Connor, Rachel Grashow, Adam Tenforde, Meagan M. Wasfy, Marc G. Weisskopf, Frank Speizer, Ross Zafonte, Aaron Baggish

**Affiliations:** 1 Football Players Health Study at Harvard University, Harvard Medical School, Boston, Massachusetts, United States of America; 2 Department of Neurology, Berenson Allen Center and Division for Cognitive Neurology, Beth Israel Deaconess Medical Center, Boston, Massachusetts, United States of America; 3 Department of Environmental Health, Harvard TH Chan School of Public Health, Boston, Massachusetts, United States of America; 4 Department of Physical Medicine and Rehabilitation, Spaulding Rehabilitation Hospital, Charlestown, Massachusetts, United States of America; 5 Department of Internal Medicine, Cardiovascular Performance Program, Massachusetts General Hospital, Boston, Massachusetts, United States of America; 6 Channing Division of Network Medicine, Department of Medicine, Brigham and Women’s Hospital, Boston, Massachusetts, United States of America; University of Florida, UNITED STATES

## Abstract

**Background:**

Participation in American-style football (ASF), one of the most popular sports worldwide, has been associated with adverse health outcomes. However, prior clinical studies of former ASF players have been limited by reliance on subjective self-reported data, inadequate sample size, or focus on a single disease process in isolation.

**Objective:**

To determine the burden of objective multi-system pathology and its relationship with subjective health complaints among former professional ASF players.

**Methods:**

The In-Person Assessment is a case-control, multi-day, deep human phenotyping protocol designed to characterize and quantify pathology among former professional ASF players. Participants, recruited from an on-going large-scale longitudinal cohort study, will include 120 men who report either no health conditions, a single health condition, or multiple health conditions across the key domains of cardiometabolic disease, disordered sleep, chronic pain, and cognitive impairment. Data will be collected from validated questionnaires, structured interviews, physical examinations, multi-modality imaging, and functional assessments over a 3-day study period. A pilot study was conducted to assess feasibility and to obtain participant feedback which was used to shape the final protocol.

**Results:**

This study provides a comprehensive assessment of objective multi-system pathology and its relationship with subjective health complaints among former professional ASF players.

**Conclusion:**

The study will determine whether subjective health complaints among former professional ASF players are explained by objective explanatory pathology and will provide novel opportunities to examine the interrelatedness of co-morbidities. It is anticipated that this protocol will be applicable to other clinical and occupational populations.

## Introduction

Adverse health outcomes among American-style football (ASF) players have received considerable attention in both the lay press and scientific literature over the past several decades. Media outlets have documented former collegiate and National Football League (NFL) players living with health conditions including chronic pain [1–5], obesity [3], cardiovascular disease [3], and cognitive deficits attributed to chronic traumatic encephalopathy (CTE) [4]. In parallel, clinical studies of former NFL players have reported cognitive impairment [6, 7], sleep-disordered breathing [8], and coronary artery calcification [9, 10]. Epidemiological data suggest that people who participate in professional ASF participation have increased risk for developing neurodegenerative disease [11], cognitive deficits [6, 12], hormonal insufficiencies [13], cardiovascular disease [14], cardiometabolic disease [15,16], chronic pain [17] and total joint replacements [18], and decreased life expectancy [19], specifically, among certain ASF field positions [20].

Fundamental limitations of prior work include the use of self-reported data without objective validation, inadequate sample size, and recruitment bias [21]. In addition, most prior clinical studies of former professional ASF players have been of limited scope with focus on specific disease processes in isolation [14–18]. This approach precludes the opportunity to examine the influence of comorbid conditions and the potential inter-relatedness of pathology across different organ systems. This limitation is of paramount importance as perceived or objective impairment may stem from primary single organ pathology (e.g. hip pain attributable to osteoarthritis) or from secondary organ system involvement (e.g. neurocognitive impairment caused by sleep pathology). Accordingly, causal relationships between professional ASF participation and long-term health remain incompletely understood. Clarification of the prevalence, severity, mechanistic underpinnings, and clinical implications of pathology among former professional ASF players is a scientific imperative. Concrete understanding of how early life ASF participation impacts later life health and wellness is required to shape rules and regulations that define the safety of ASF and to inform opportunities for disease prevention and management. Lessons learned from the rigorous study of former professional ASF athletes may also be generalizable to other populations including the military and other contact sport athletes.

The Football Players Health Study (FPHS) at Harvard University, established in 2014, was designed to address key scientific uncertainties regarding ASF participation and long-term health. The FPHS integrates broad scale epidemiology with deep clinical phenotyping [22]. The clinical phenotyping protocol, termed the In-Person Assessment (IPA) study, compares self-reported health status with objective gold standard clinical measurements across the fundamental domains of 1.) neurocognitive function, 2.) cardiometabolic health, 3.) musculoskeletal function/pain and 4.) sleep quality. The IPA was designed to determine relationships between subjective health affliction and objective pathology and to examine the interdependence of diseases across discrete organ systems. We anticipate the resultant findings will contribute to understanding mechanisms of illness and guide future clinical work dedicated to disease treatment and prevention. The methodology of the IPA constitutes a distinctly novel, readily transferable and highly productive approach to in-depth, multidisciplinary clinical phenotyping. As IPA recruitment coincided with the COVID-19 pandemic, our model also uniquely demonstrates how research can continue effectively during an infectious disease pandemic while ensuring the safety of participants and research personnel. Here, we present the IPA methodologic approach as it may serve as a model for application in other clinical and elite athletic populations.

## Methods

### Study design overview

**Fig 1:**
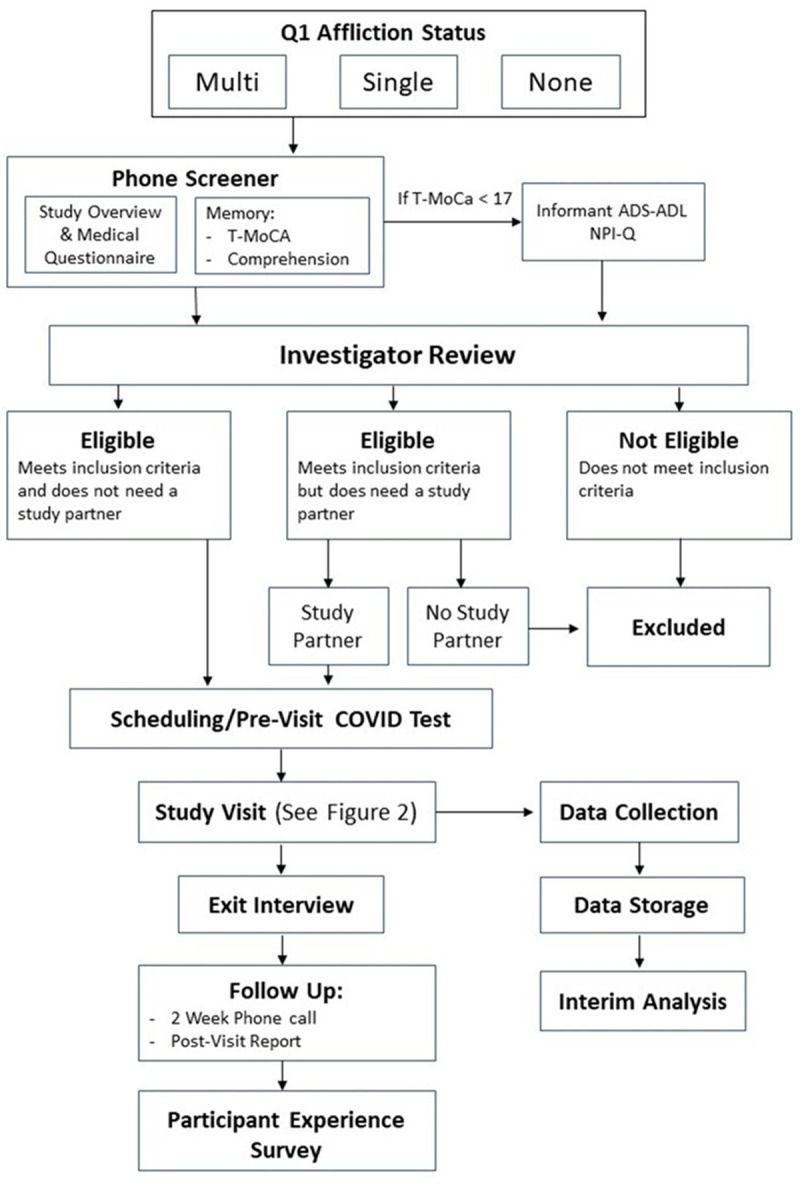
Flow diagram depicting study design of the Football Players Health Study’s In-Person Assessment.

The IPA is a, multi-day, in-person study of physical, behavioral, and physiological health in former professional ASF players (Fig 1). Potential IPA participants are recruited from a large, ongoing epidemiologic cohort study comprised of former professional ASF players who have completed a comprehensive baseline health questionnaire. Data derived from this questionnaire are used to identify men with subjective affliction (i.e. “cases”) and men without affliction (“controls”) across the principal four health domains of neurocognitive function, cardiometabolic heath, physical function/pain, and sleep quality [15]. Eligible participants are categorized as afflicted in one health domain, afflicted in multiple health domains, or unafflicted. Eligible participants meeting criteria for one or multiple afflictions are “matched” with unafflicted eligible participants of similar age and race. All eligible participants complete a phone screening to assess for willingness to participate, capacity to consent, and for the need to have assistance with travel, study enrollment, and/or study completion. Informed consent is obtained either in-person or virtually for all participants prior to study enrollment. Once recruited, enrolled participants complete a battery of 12 discrete assessments over a three-day period which integrate the clinical domains of neurology, psychiatry, cardiovascular medicine, sleep medicine, orthopedics, pain medicine, physical medicine and rehabilitation, otorhinolaryngology, and endocrinology. Upon study completion, participants undergo a structured exit interview during which they receive verbal and written results for clinically relevant metrics with established general population reference data (e.g., total cholesterol, body composition). A designated study nurse and physician are available to help participants follow up with their primary care providers or identify specialists for any individual results that may require further evaluation and treatment. All aspects of this study and the preparatory pilot study are conducted at Harvard Medical School affiliated hospitals and have been approved by the Mass General Brigham Human Subjects Research Committee (Protocol # 2018P001929 and Protocol # 2017P000139, respectively).

### Recruitment, screening, and participant matching

Recruitment and inclusion criteria for the IPA are linked to the epidemiology arm of the FPHS. In 2014, FPHS launched a baseline self-completed health questionnaire (Q1). To be included in the Q1 cohort and potentially eligible for the IPA, a participant must be a former professional football player who played in the American or National Football League after 1960. Former players of all ages, races, and home geographic locations were eligible. To date, over 4,000 former players have completed Q1 [21]. The degree to which the FPHS Q1 cohort is representative of the entire pool of former players has been assessed using publicly available and licensed game, position and starter data for all NFL players who played at least one NFL game. As previously reported, the FPHS Q1 cohort is well matched to the entire pool of former players based on field position distribution, years played, reported playing weight and height [23].

Former players are identified as potential participants for the IPA based on the presence or absence of self-reported affliction in key domains of affliction include chronic pain, sleep apnea, cardiometabolic disease, and cognitive impairment. These four categories were chosen based on data from Q1 and prior research [13–15, 23–25]. Definitions for these health afflictions are presented in Table 1 [15, 24]. Eligible IPA participants with none of these afflictions are defined as “unafflicted”. Eligible IPA participants with affliction(s) limited to a single category are defined as “single-afflicted” and those with afflictions in ≥3 categories are defined as “multi-afflicted”. Q1 participants with exactly two afflictions were excluded to maximize separations between affliction categories. To minimize recruitment bias and to maximize the generalizability of our results across the entire former player community, for each “multi-afflicted” eligible participant, a potential match, based on race and age ±2 years, in either the “single-afflicted” or “unafflicted” group is identified. Potential “multi-afflicted” participants are contacted by email or telephone call as described below. When a “multi-afflicted” participant agrees to enroll and their eligibility status is confirmed, their participant match(es) are similarly contacted. Based on *a priori* power calculations and to account for attrition, we aim to recruit 50 participants in each of the above categories with a goal of 120 participants completing the full study. At time of study protocol journal submission (September 8th, 2021), 77 participants have been screened for eligibility and 54 participants have completed the full study. It is anticipated that this study will last approximately 2.5 years.

**Table 1:** Definitions used for health afflictions in the In-Person Assessment study.

Affliction Category					
**Cardiometabolic Disease**	Self-report of having had a healthcare provider diagnose heart attack or stroke	OR	Having ever been prescribed or currently taking medications for two of the three following conditions:	OR	Having undergone a cardiac related surgery.
			• hypertension		
			• diabetes		
			• high cholesterol		
**Chronic Pain**	Self-report of chronic pain	AND	Current management of chronic pain by either over the counter or prescription medication or alternative therapies		
**Neurocognitive Impairment**	Self-reported health care diagnoses of Alzheimer’s Disease/dementia	OR	Self-reported health care diagnoses of chronic traumatic encephalopathy	OR	Having ever been prescribed or currently taking medication for memory loss
**Sleep Disorders**	Self-report of having had a healthcare provider diagnose sleep apnea				

All potential participants complete a phone screening with a trained study staff member to determine eligibility status. This includes questions regarding the participant’s medical history, a cognitive screening assessment using the Telephone Montreal Cognitive Assessment (T-MoCA) [26, 27], and confirmation that the participant demonstrates understanding of study goals and procedures. If cognitive impairment is suspected, additional information is gathered from an individual in close relation to the potential participant (i.e., spouse, healthcare proxy, etc.) to further assess the potential participant’s functional status using the Alzheimer’s Disease Cooperative Study (ADCS-ADL) [28] Inventory and the Neuropsychiatric Inventory Questionnaire (NPI-Q) [29]. One of the study investigators reviews the cognitive screening and determines if the participant would 1) need to travel with a study partner to act as a legally authorized representative for consent or, 2) if the participant is too impaired functionally or behaviorally due to their cognition to take part in the study. An incentive stipend of $600 per each full day of participation totaling $1800 is offered to all participants as approved by the IRB.

#### Inclusion & exclusion criteria

IPA inclusion criteria are summarized as follows. First, IPA eligibility is limited to men who were between 24 to 55 years of age at the time of Q1 completion. This age restriction is utilized to minimize the impact of aging which is an established risk factor for many of the disease processes under investigation. Second, to ensure successful participant matching, eligibility is further confined to men who self-identify and self-report race as either ‘Black/African American’ or ‘White’ as only a small proportion of former NFL participants who self-identify as other races and ethnicities. Finally, IPA eligibility is confined to men who were Q1 completers before January 1, 2017. This restriction was enacted to ensure IPA participants completed Q1 a minimum of two years prior, thus making them eligible for the follow-up questionnaire, the Second Health and Wellness Questionnaire (Q2), which queries additional ASF exposure and self-reported health data. Potential participants are excluded if they are deemed to have significant neurocognitive impairment *and* an inability to have a legally authorized representative participate in the informed consent process. In addition, potential participants are excluded if they are unable or unwilling to undergo the brain MRI portion of the study at time of study enrollment (due to claustrophobia, non-removable metal implants, etc.).

### Data collection

Data collection for the IPA begins with a comprehensive review of Q1-reported ASF exposure variables hypothesized to be previously linked to health outcomes [15, 22, 24, 30], including number of years of AFL/NFL football play [25, 31], number of AFL/NFL games played [31], field position differentiating linemen and non-linemen [32, 33], and number of concussions sustained in both AFL/NFL and non-AFL/NFL leagues (including high school and collegiate) [12]. Each of these putative risk factors is measured via self-reported data and the first three are confirmed using corresponding public data from the National Football League and Pro-Football Reference [34]. In addition to these conventional ASF exposure metrics, numerous additional ASF-related and non-ASF related exposure variables are assessed (Table 2).

**Table 2:** American-style football exposure metrics and non-American-style football exposure metrics measured in the Football Players Health Study’s In-Person Assessment.

Play Related Exposures	Non-Play Related Exposures
Number of Years Played Before High School	Dietary and Nutritional Supplements During Professional Career
Number of Years Played During High School	Pain Medication and Other Drugs During Professional Career
Positions Played During High School	Use of Caffeine to Improve Sport Performance
Number of Years Played During College	Use of Energy Drinks to Improve Sport Performance
Positions Played During College	Use of Creatine to Improve Sport Performance
Number of practices during college including preseason and regular season per week	Use of Steroids to Improve Sport Performance
Number of practices during college including preseason and regular season per week that were in full pads or shoulder pads	Use of Growth Hormone or Insulin-life Growth Factor to Improve Sport Performance
Number of snaps per game played at each position during college	Use of Ephedra to Improve Sport Performance
Age of Onset for Football Participation	Use of Beta-Hydroxy Beta-Methylbutyrate to Improve Sport Performance
Number of Diagnosed Concussions	Use of Non-Caffeine Stimulants to Improve Sport Performance
Frequency of mild to severe concussion symptoms (e.g., dizziness, confusion, nausea, seizure) experienced after a blow to the head or neck	
Number of Years Played Professionally	Use of Red-Cell Boosting Agents or Techniques to Improve Sport Performance
Positions Played Professionally	Use of Other Cardiovascular Enhancement Agents to Improve Sport Performance
Number of practices during NFL career including preseason and regular season per week	
Number of practices during NFL career including preseason and regular season per week that were in full pads or shoulder pads	
Number of snaps per game played at each position during NFL career	
Additional non-NFL professional football exposure	

**Fig 2:**
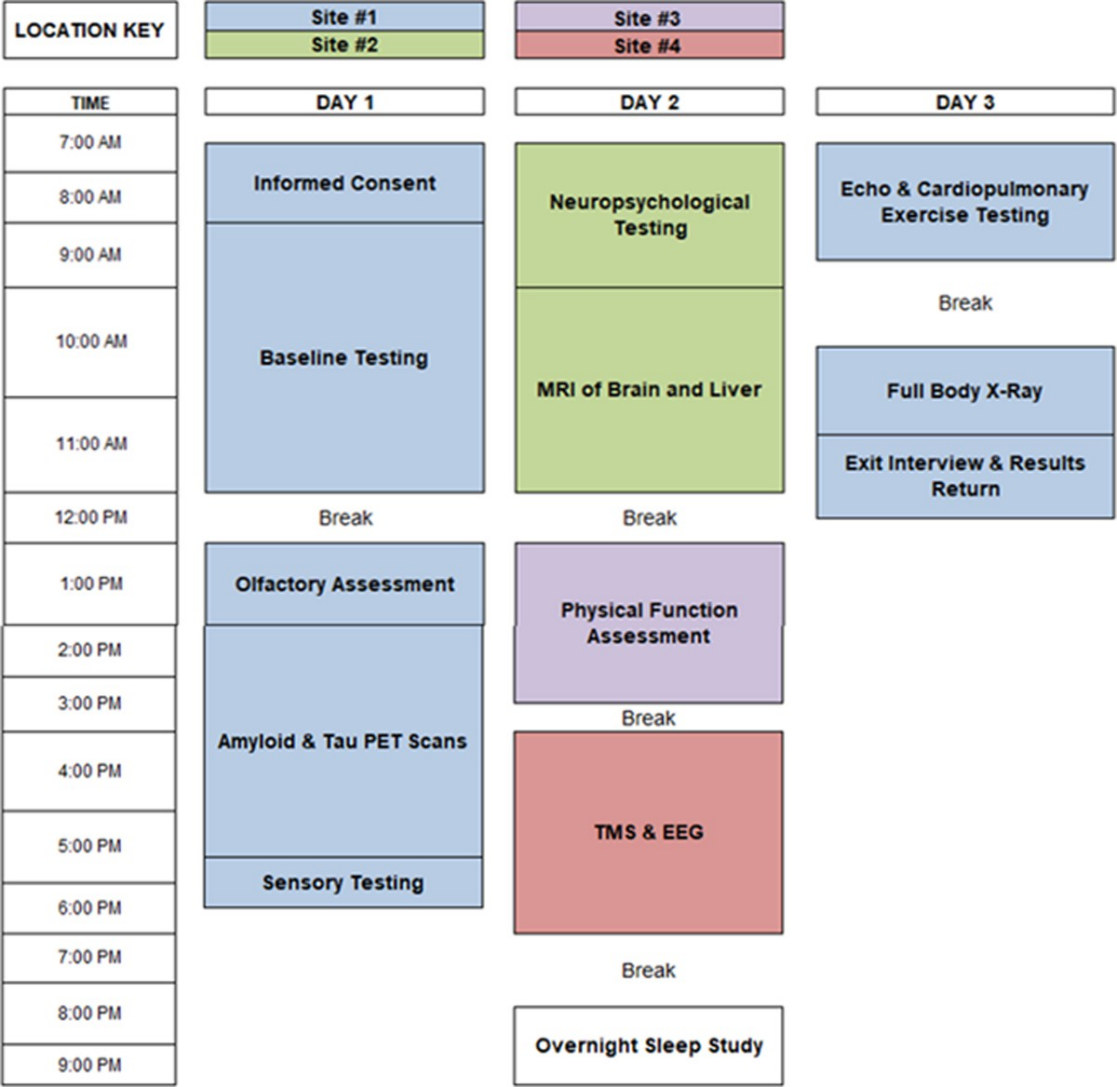
Daily participant testing protocol for the Football Players Health Study’s In-Person Assessment.

Each IPA participant completes 12 “blocks” of testing over three days across four different hospital sites (Fig 2). Testing blocks include self-completed questionnaires, structured interviews, physical examinations, multi-modality imaging, and functional assessments. For example, the “Baseline Assessment” block includes: a dual-energy x-ray absorptiometry (DXA) scan, fasting blood samples to ascertain metabolic and hormone panels, an oral glucose tolerance test, collection of vital signs (blood pressure, heart rate, etc.), anthropometric measurements, and validated questionnaires including the International Physical Activity Questionnaire. Subsequent testing blocks include amyloid & tau positron emission tomography scans, olfactory assessment, sensory testing, neuropsychological testing, magnetic resonance imaging (MRI) of the brain and liver, physical function assessment, transcranial magnetic stimulation (TMS) and electroencephalogram (EEG), overnight sleep study, cardiopulmonary exercise testing, transthoracic echocardiography, radial artery applanation tonometry, and full body x-ray imaging (S1 Appendix). Participants are escorted to and from all testing blocks by a medical staff navigator, to ensure on time arrival and testing block completion.

### Return of results

Upon study completion, laboratory results and other clinically relevant data that are available by the end of the three-day study visit are provided to participants during a “return of results” meeting conducted by a study physician and the medical navigation nurse (Table 3). The “return of results” meeting is structured to educate the participant about pertinent, clinically actionable findings. With permission, consisting of written HIPAA release form, relevant results are also sent to the participant’s primary care provider or designated sub-specialist(s) to facilitate subsequent additional diagnostic testing and clinical management. Participants with incidental findings that merit urgent additional assessment and/or treatment are offered these services at one or more of the participating hospitals. Participants are also provided by mail with a post-visit report containing the results of testing for which clinical interpretations were not available for presentation during the structured “return of results” meeting due to processing time.

**Table 3:** Clinical results returned to participants in the Football Players Health Study’s In-Person Assessment during the Exit Interview (A) and in the Post Visit Report* (B).

Assessment	Report
**A. Exit Interview**	
**Vital signs and anthropometrics**	Values collected at baseline
**Dual-energy x-ray absorptiometry (DEXA) Scan**	Highlights of the scan are shared with easy-to-understand descriptions of the meaning of the results
**Clinical blood testing**	Results of all values are provided in the report with a brief description of the meaning of the test. (See details of tests performed in S1 Appendix).
**Brain and liver magnetic resonance imaging**	A clinical report is reviewed with the participant and a copy is provided.
**Neuropsychiatric testing**	A selection of health and well-being questionnaire and cognitive test results are provided along with easily understood descriptions of these results. If there are concerns for an active issue that would benefit from follow-up, this is shared with the participant.
**B. Post Visit Report 6–8 weeks later**	
**Sensory Testing–(QST)**	Testing of pain tolerance, sensitivity, temporal summation, and inhibition are provided in a simple scale of: at, above, or below average.
**Liver MRI**	Reports percentage of liver fat
**Physical Function**	Selective Functional Movement Assessment (SFMA): Chart of mobility in various body regions
	Sit to rise: Overall score with age ranges
	Y-Balance test: Measures of asymmetry and leg reach
	6 Minute Walk Test: Chart of speed and distance with normal ranges
	Stair Climb Power: Score with normal ranges by age
	Muscle Performance: measures of strength, power, and endurance
	Reaction Time: chart of reaction and movement time
**Dual Task**	Measure of postural sway, fall risk, and dual task cost
**Sleep Assessment**	Apnea/Hypopnea Index with ranges of normal to severe
**Cardiovascular Testing**	Cardiopulmonary exercise test: Resting metabolic rate and peak exercise capacity
	Echocardiogram: Indices of heart muscle and valve structure and function
**X-ray**	Measures of joint arthritis with ranges from normal to severe
**Electroencephalogram**	Notification of any abnormalities from assessment with ability to share full report with participants doctor

### Data privacy, collection, and storage

Numerous precautions are taken to ensure that all IPA data are maintained in private and secure fashion. All staff members receive training to work with highly confidential information and abide by strict medical and ethical standards. To ensure confidentiality and privacy, all study participants are assigned a unique study identification number that is connected to their identifiable information through a secure and limited-access database available only to trained study staff. All study data are submitted from participating sites using encrypted data transfer options in compliance with guidelines from Mass General Brigham and Harvard Medical School. In addition, all data obtained are stored in secure, custom‐built data repositories that are comprised of servers that are encrypted at rest. All identifiable data are coded and removed from any working files. Finally, a Certificate of Confidentiality was obtained from the National Institute of Health (NIH) prior to enrollment commencement as an added layer of data protection.

### Statistical considerations

Power calculations accounted for various proportions of afflicted participants and were found to have at least 80% power to measure associations at the 0.05 level of significance between our affliction categories and multiple outcomes of interest including neuropsychological and cardiovascular outcomes. We anticipate little missing data due to the in-person nature of the visit, and the in-depth screening required before participants are recruited, enrolled and arrive for the study visit. For example, potential participants with claustrophobia that would prevent MRI or PET participation are not enrolled in the IPA. To the extent that individual tests or outcomes may be missing (e.g. refusing parts of the physical function exam due to chronic orthopedic pain) will be addressed by assigning missing data indicators and including predictors of missingness in our models. We expect remaining missing data (machine failure or unavailability) to be “missing completely at random” and therefore unlikely to significantly bias estimates of association. We will initially explore relationships using univariate analyses. Linear and nonlinear relationships between exposures and outcomes in multivariate adjusted models will be investigated. To account for selection in statistical analyses, we will use methods including restriction, stratification, and inverse probability weighting. Finally, generalizability will be assessed by comparing the personal characteristics of those participating in the IPA with the larger cohort from which they were selected.

### Pilot study

To examine the logistical feasibility and patient tolerability of the IPA, a pilot study was conducted. The pilot study consisted of two discrete stages, each with a total enrollment of 10 men (age range = 24–55 years) with no prior ASF exposure. Stage 1 included five “healthy” or non-afflicted participants and Stage 2 consisted of five participants with health complaints and body habitus similar to the former ASF player population (height = 70.9 +/- 5.2 inches, weight = 271.6 +/- 73.2 lbs.), aged 24–55. Stage 2 participants underwent a T-MoCA to assess for mild cognitive impairment. Participants were recruited from the local community using the Partners Research Study Volunteer Program (RSVP) for Health and participant eligibility was determined by telephone screening.

The pilot study protocol involved four days of assessments. The first two days contained “core” clinical assessments (e.g., overnight sleep study, cardiometabolic testing, neuropsychological evaluation) and days 3 and 4 contained additional assessments/mock assessments that were investigational in nature [e.g., transcranial magnetic stimulation (TMS), positron emission tomography (PET)/computed tomography (CT), etc.]. PET/CT, DXA, and x-ray assessments were conducted in a “mock” manner in the pilot study so that participants did not receive any ionizing radiation. Following the completion of the pilot study, all participants were asked to provide comprehensive feedback to assist with the modifications of the study design and logistics. Participant feedback coupled with input from the investigative team and the FPHS Player Advisors, a group of former professional ASF players that offer perspectives on best practices, informed the final study design of the IPA. Feedback from the pilot study resulted in several key protocol changes that included a reduction the protocol duration from four to three study days, increased frequency of scheduled participant rest breaks, and a standardization of meals based on participant’s body mass.

### Interim analysis

As part of the IPA protocol, a pre-specified interim analysis of data from all clinical assessments was scheduled to be performed after completion of the 30^th^ former player participant. The goal of this interim analysis was to confirm successful data capture across the key assessment domains. Preselected key outcome variables derived from each assessment domain were assessed using descriptive statistics. Group values between subjects who were characterized as unafflicted (n = 9), single afflicted (n = 11), and multi-afflicted (n = 10). Summary data emerging from this basic assessment using non-parametric statistics to examine trends coupled with a report of missing data points were provided to each disease domain co-investigator with a goal of identifying any systematic errors.

### Participant satisfaction

Each participant upon completion of the IPA is provided with the Participant Satisfaction Survey, designed to assess satisfaction with the research experience and any short-term action items that arose following their completion of the IPA (S2 Appendix). This survey queries topics including overall study experience, experiences with hospital and study staff members, utilization of medical results, and post-IPA follow up with community clinical care providers. Of the 47 participants who have participated in the study, 27 have completed the survey (67.5% response rate). All participants “agreed” or “strongly agreed” that they were “satisfied with their study experience” and 92% “agreed” that their “expectations of the study matched their experience”. 96% of participants “strongly agreed” that FPHS staff members were knowledgeable, friendly, and helped make the study a positive experience. Each participant indicated that they had referred to their post-visit results report and 88% contacted the medical navigation nurse in the context of the Participant Satisfaction Survey, to address outstanding questions. All but one participant indicated that they had learned something new about their health from the IPA and 77% followed up with a specialist after their visit. The medical navigation nurse assisted 44% of the participants with finding medical subspecialists in their local communities to address conditions detected during the IPA. 78% of participants noted implementing specific lifestyle changes based on their personal IPA results.

### Continuing IPA research during Covid-19

In response to Covid-19 pandemic, the IPA was temporarily halted in mid-March 2020. Previously scheduled visits were postponed to a later date and enrollment and recruitment were suspended. During this break, precautions to ensure the safety of participants, study staff, and hospital staff were structured and implemented. After a 5-month hiatus, the IPA resumed research activity in September 2020. Initial participant recruitment focused on local potential participants who could drive to Boston from states that were considered “low-risk”, as outlined by the commonwealth of Massachusetts Covid-19 Travel Order. The recruitment catchment was slowly increased as more states became low risk due the dissemination of vaccine and as increasing scientific data demonstrating safety of airline travel became available.

The IPA’s safety guidelines and precautions for return to research incorporated regulations set by Mass General Brigham and its affiliates, public health guidance provided by the Centers for Disease Control and Prevention (CDC), and regulations delineated by the Massachusetts Department of Public Health. Additional safety precautions beyond those outlined by the above entities were implemented after discussion with IPA study investigators, FPHS Player and Family Advisors, and FPHS study staff. Specific measures implemented in response to the COVID-19 pandemic included: 1) daily symptom attestation and weekly COVID-19 PCR testing for all IPA study staff who travel with participants between study sites, 2) participant COVID-19 PCR testing within 72 hours prior to arrival and within 1 week of study completion, 3) provision of a personal supply of face masks, hand sanitizer, and disinfecting wipes for each participant upon arrival to Boston, Massachusetts, USA, 4) provision of educational material about hospital public health measures including hand hygiene, social distancing and disinfection protocols. In addition, all facilities used during the IPA including participant waiting rooms, clinical assessment rooms, and eating facilities were reconfigured to ensure a minimum physical distancing of six feet.

### Research challenges and mitigation strategies

While the IPA protocol was carefully reviewed by all relevant regulatory bodies and the study funder, residual human subject research challenges are anticipated. The 2.5-day IPA study includes assessments in 12 clinical domains across four hospital facilities which requires the coordination of many scientific investigators, administrative staff, and lab personnel. Accordingly, unanticipated “day of visit” challenges including technical issues with equipment, laboratory closure due to staff illness or absence, and claustrophobia during brain MRI are unavoidable. The impact of these and other potential disruptions are minimized by our use of an IPA navigation team that escorts participants between study sites and communicates with study personnel about all potential protocol interruptions. This approach facilitates real time schedule alterations that maximize the opportunity to complete all aspects of the study thereby minimizing missing data. Despite these efforts, we anticipate some participants may have incomplete or uninterpretable data and have set conservative sample size targets which will maximize our final analytic power to detect statistically and clinically significant across group differences.

We also anticipate challenges with recruitment, particularly during the remainder of the COVID-19 pandemic. While we typically enroll and study one participant per week, our protocol design allows for enrollment of two participants per week by staggering tests over five hospital weekdays of operation to ensure we meet target recruitment. To further optimize recruitment, we designed the IPA to make participation appealing for potential participants by including appropriate financial compensation, transportation for both the participant and family member to travel to Boston to complete the assessment, and return of clinically relevant results from IPA to the participant at conclusion of the visit. This personalized report which contains study results and constructive health information in basic language, easily understood by participants with little or no medical background, has been particularly valued by participants and their families.

## Conclusion

The FPHS IPA was designed to evaluate the relationship between subjective health affliction and objective clinical pathology in former ASF players. Utilizing a novel, multi-day, deep phenotyping protocol, the IPA collects objective data defining health, wellness, and pathology with an emphasis on the key domains of cardiometabolic disease, sleep quality, chronic pain, and cognitive impairment. Importantly, simultaneous assessment of multiple health domains provides an opportunity to explore the interrelated nature of concurrent co-morbidities with an ultimate goal of defining previously unidentified causal mediators of health and affliction.

To our knowledge, the IPA is the first study to examine “whole body” health and affliction in former ASF athletes. It is anticipated that a “whole body” perspective will provide novel insights into how one seemingly discrete health condition can impact other types of pathology and examine the interrelationship of injury and disease. For example, sleep apnea, a common issue in former ASF athletes, is a known risk factor for and causal mediator of neurocognitive impairment [35] and cardiovascular disease [36]. At present, the degree to which clinically relevant cognitive impairment and cardiovascular pathology are driven by disordered sleep rather than intrinsic dysfunction of the brain, heart, and blood vessels remains incompletely understood. By combining polysomnography data with neurocognitive and cardiovascular testing results in former ASF players, we anticipate the opportunity to establish new causal relationships and subsequent targets for prevention and intervention.

In summary, this paper presents a study design and methodology for validating subjective self-reported health data with objective clinical assessments in a population susceptible to substantial workplace hazards. The IPA study represents a new paradigm to better examine the relationship between occupational health exposures and health outcomes. The rationale and approach upon which the IPA was designed may be applicable to other at-risk populations including emergency responders [37], military veterans [38], boxers, and other contact sport athletes [39]. Furthermore, by studying both direct occupational exposures and indirect occupational hazards we hope to create a more robust framework for future research on both ASF players and other populations. Similarly, by examining relationships between comorbidities, we aim to shift the future of ASF former player research from a single-disease focus to one that emphasizes multi-system biology.

Because this effort is ongoing and the methods being used are relatively novel, we present the design and methodology of the FPHS IPA in recognition of the fact that our methodology and guiding scientific principles may serve as templates for future studies of other discrete populations.

## Supporting information

S1 Appendix In-Person Assessment Blocks. (DOCX)

S2 Appendix Participant Satisfaction Survey. (DOCX)
