# Supplementary material for: Multi-modality human phenotyping to examine subjective and objective health afflictions in former professional American-style football players: The In-Person Assessment (IPA) protocol
Source: PLoS One. 2022 Mar 31;17(3):e0265737. doi: 10.1371/journal.pone.0265737 (PMC8970522; doi:10.1371/journal.pone.0265737)
Supplement: S1 Appendix — (DOCX) [file pone.0265737.s001.docx]

**S1 Appendix. In-Person Assessment Blocks.**

| **Baseline** | |
| --- | --- |
| Medical History and Medication Review  Physical Exam  Orthostatic vital signs  Anthropometrics  Height and weight | Fasting Laboratory Samples:   - Comprehensive Metabolic Panel, HbA1c, Insulin - Lipids and lipoproteins - Hormones - Complete Blood Count - Liver Function tests - Thyroid function tests - Oral glucose tolerance test |

| **Assessments** | | |
| --- | --- | --- |
| Dual-energy x-ray absorptiometry (DXA) | The identified protocol is aimed at measuring whole body, hip, and AP spine**,** and regional fat; and muscle |  |
| Otorhinolaryngologic assessment | The olfactory test battery is comprised of three different measures of olfactory function: odor identification, episodic olfactory memory, and odor discrimination | The olfactory neural system is particularly vulnerable to AD pathology [40,41]. Reduced odor identification after, but not before, 33 months from their last concussion [42]. |
| PET/CT imaging | Amyloid PET imaging conducted using C-11PiB  Tau PET imaging using F-18 T807 (Flortaucipir) | C-11 PiB, specific for imaging amyloid plaques and F-18 T807, for imaging tau accumulation, will be used to define the prevalence of cerebral amyloid and tau protein [43-45] . |
| Quantitative Sensory Testing | Quantitative Sensory Testing (QST) is a method for diagnosing peripheral nervous system disorders, including chronic pain and pain related to various diseases[46,47]. | The QST battery will provide a comprehensive assessment of pain sensitivity and pain-modulatory capacity |
| Magnetic Resonance Imaging (MRI) | A comprehensive Brain MRI will be completed including resting state functional magnetic resonance imaging (fMRI), diffusion tensor imaging (DTI), arterial spin labeling (ASL), susceptibility weighted imaging (SWI), fluid-attenuated inversion recovery (FLAIR), single voxel spectroscopy, and chemical shift imaging (CSI) | The MRI will gather a comprehensive, objective assessment of brain structure and function. Focus will be on assessing gray matter integrity, white matter connectivity, functional connectivity, brain perfusion, and brain metabolism |
|  | An MRI of the liver will be completed using the Dixon/PDFF technique to that will provide contrast between fat versus non-fat tissues | The liver MRI will be used to assess for hepatic steatosis as an additional metabolic assessment |
| Physical Function Assessment | The physical function battery consists of the following assessments: The Selective Functional Movement Assessment (SFMA - Functional Movement Systems, Lynchburg, VA) [48,49]. Muscle Strength Testing [50,51], Muscle Endurance Testing [52], Muscle Power Testing, Sitting-Rising Test [53,54], a Loaded Stair Climb [55,56], the Six-Minute Walk Test (6-MWT) [57], the Y-Balance Test [58], Choice Stepping Reaction Time (CSRT) [59], and a Dual Task Assessment | The physical function assessment aims to collect information about muscle performance, physical function, gait, balance, and cognitive reserve via a dual-task assessment |
| Transcranial Magnetic Stimulation (TMS) and EEG | Transcranial Magnetic Stimulation and EEG will be used to measure Intracortical Inhibition and Facilitation Using Paired and Single TMS Pulses to identified cortical regions. Baseline EEG Measures will be captured to assess resting state and task provoked responses. | TMS measures have been shown to reveal physiologic biomarkers of brain cortical dysfunction related to acute concussions and history of past concussions [60,61]. These measures may relate to cognitive function and represent early indicators of risk of cognitive decline |
| Sleep Study | Polysomnography (PSG) will be conducted overnight. A Psychomotor Vigilance Test (PVT) will be conducted | This sleep assessment aims to collect information that will help to better understand the relationships between sleep disturbances and health outcomes including hypertension, diabetes, and heart diseases (such as heart attack, stroke, and heart failure) |
| Cardiac Assessment Battery | The cardiac assessment battery includes:   - Transthoracic Echocardiography (TTE) - Resting Metabolic Rate (RMR) - Cardiopulmonary Exercise Test (CPET) on upright cycle ergometer - Arterial Tonometry for arterial stiffness using applanation tonometer | Participation in American-style Football has been associated with numerous maladaptive cardiovascular attributes including hypertension [62], vascular stiffening [62], cardiac hypertrophy [63], and increased mortality [64]. A primary objective of this protocol will be to develop a comprehensive cardiovascular phenotype profile for former professional football athletes |
| X-Ray Imaging | The X-Ray imaging will simultaneously obtain standing anteroposterior and lateral views of the spine (scoliosis series), lower extremities (joint survey), and whole body. | These X-ray images of the shoulders, ankles, knees, hips and spine will be correlated with potential physical impairments to better understand and quantify functional limitation and pain |
|  |  |  |

| **Neuropsychological and Cognitive Testing** |
| --- |
| The NIH Toolbox Cognition Battery |
| - Picture Vocabulary - Oral Reading Recognition - List Sorting Working Memory - Picture Sequence Memory - Pattern Comparison Processing Speed - Flanker Inhibitory Control and Attention - Dimensional Change Card Sort |
| **Traditional Neuropsychological Assessment Battery** |
| - Advanced Clinical Solutions Test of Premorbid Functioning - Reynolds Intellectual Screening Test (Guess What and Odd Item Out) - TOMM Trial 1 (Trial 2, only if necessary; a Performance Validity Test) - Neuropsychological Assessment Battery (NAB): Language Module, Naming; Memory Module, List Learning, Story Learning, and Daily Living Memory - Rey Complex Figure (Copy Trial Only) - Delis-Kaplan Executive Function System: Color Word and Verbal Fluency |
| **Cognitive Neuroscience Battery [65]** |
| - Gradual Onset Continuous Performance (gradCPT) – a test of inhibition - Serial Reaction Time Test [66] |
| **MINI Neuropsychiatric Interview** |
| **Battery of Psychological Health and Quality of Life Outcome Measures** |
| - PHQ-9: Patient Health Questionnaire - CHRT: Concise Health Risk Tracking Scale - AUDIT: Alcohol Use Disorders Identification Test - NIDA: National Institute on Drug Abuse screen - GAD-7: Generalized Anxiety Disorder questionnaire - Neuro-QoL Emotional and Behavioral Dyscontrol - PSS: Perceived Stress Scale - ACE: Adverse Child Experience questionnaire - PROMIS Pain Interference 8-item short form - Post-Concussion Scale - PROMIS Satisfaction with Participation in Social Roles - NIH Toolbox Meaning and Purpose - Sheehan Disability Scale (SDS) - Gratitude Questionnaire - Connor-Davidson Resilience Scale (CD-RISC) - Injustice Experience Questionnaire |

| **Questionnaires** | |
| --- | --- |
| Baseline | - International Physical Activity Questionnaires (IPAQ) - Pain Catastrophizing Scale - Sexual health inventory for men (SHIM) |
| Sleep | - PROMIS: Sleep Disturbance Scale – short form - PROMIS: Sleep Impairment Scale – short form - PROMIS: Fatigue - Functional Outcomes of Sleep Questionnaire (FOSQ) - Ford Insomnia Response to Stress Test (FIRST) - Multi-Ethnic Study of Atherosclerosis (MESA) Sleep Questionnaire – selected questions to further asses sleep timing and restless leg syndrome - Women’s Health Initiative Insomnia Rating Scale (WHIIRS) - Munich Chronotype Questionnaire (uMCTQ)- short form - Sleep Heart Health Morning Survey |
| Physical Function | - Knee injury and osteoarthritis outcome score (KOOS) - Hip dysfunction and osteoarthritis outcome score (HOOS) - Oswestry Disability Index (ODI) |

- 1. References

| 40. | Kovács T, Cairns NJ, Lantos PL. Olfactory centres in Alzheimer’s disease: olfactory bulb is involved in early Braak’s stages. Neuroreport. 2001;12(2):285–8. |
| --- | --- |
|  |  |
| 41. | Albers MW, Gilmore GC, Kaye J, Murphy C, Wingfield A, Bennett DA, et al. At the interface of sensory and motor dysfunctions and Alzheimer’s disease. Alzheimers Dement. 2015;11(1):70–98. |
|  |  |
| 42. | Charland-Verville V, Lassonde M, Frasnelli J. Olfaction in athletes with concussion. Am J Rhinol Allergy. 2012;26(3):222–6. |
|  |  |
| 43. | Hyman BT, Phelps CH, Beach TG, Bigio EH, Cairns NJ, Carrillo MC, et al. National Institute on Aging-Alzheimer’s Association guidelines for the neuropathologic assessment of Alzheimer’s disease. Alzheimers Dement. 2012;8(1):1–13. |
|  |  |
| 44. | Mitsis EM, Riggio S, Kostakoglu L, Dickstein DL, Machac J, Delman B, et al. Tauopathy PET and amyloid PET in the diagnosis of chronic traumatic encephalopathies: studies of a retired NFL player and of a man with FTD and a severe head injury. Transl Psychiatry. 2014;4(9):e441. |
|  |  |
| 45. | Gardner RC, Possin KL, Hess CP, Huang EJ, Grinberg LT, Nolan AL, et al. Evaluating and treating neurobehavioral symptoms in professional American football players: Lessons from a case series. Neurol Clin Pract. 2015;5(4):285–95. |
|  |  |
| 46. | Gerecz-Simon EM, Tunks ER, Heale JA, Kean WF, Buchanan WW. Measurement of pain threshold in patients with rheumatoid arthritis, osteoarthritis, ankylosing spondylitis, and healthy controls. Clin Rheumatol. 1989;8(4):467–74. |
|  |  |
| 47. | Edwards RR, Sarlani E, Wesselmann U, Fillingim RB. Quantitative assessment of experimental pain perception: multiple domains of clinical relevance. Pain. 2005;114(3):315–9. |
|  |  |
| 48. | Glaws KR, Juneau CM, Becker LC, Di Stasi SL, Hewett TE. Intra- and inter-rater reliability of the selective functional movement assessment (sfma). Int J Sports Phys Ther. 2014;9(2):195–207. |
|  |  |
| 49. | Goshtigian GR, Swanson BT. Using the Selective Functional Movement Assessment and regional interdependence theory to guide treatment of an athlete with back pain: A case report. Int J Sports Phys Ther. 2016;11(4):575–95. |
|  |  |
| 50. | Baechle TR, Earle R. Essentials of strength training and conditioning. 3rd ed. Champaign, IL: Human Kinetics; 2008. |
|  |  |
| 51. | Knuttgen HG, Kraemer WJ. Terminology and measurement in exercise performance. J Strength Cond Res. 1987;1(1):1. |
|  |  |
| 52. | Storer TW, Magliano L, Woodhouse L, Lee ML, Dzekov C, Dzekov J, et al. Testosterone dose-dependently increases maximal voluntary strength and leg power, but does not affect fatigability or specific tension. J Clin Endocrinol Metab. 2003;88(4):1478–85. |
|  |  |
| 53. | Lira VA, Araujo CGS de. Teste de sentar-levantar: estudos de fidedignidade. Rev Bras Ciênc Mov. 2000;8(2):9–18. |
|  |  |
| 54. | Brito LBB de, Ricardo DR, Araújo DSMS de, Ramos PS, Myers J, Araújo CGS de. Ability to sit and rise from the floor as a predictor of all-cause mortality. Eur J Prev Cardiol. 2014;21(7):892–8. |
|  |  |
| 55. | LeBrasseur NK, Bhasin S, Miciek R, Storer TW. Tests of muscle strength and physical function: reliability and discrimination of performance in younger and older men and older men with mobility limitations: The reliability of measures of strength and function. J Am Geriatr Soc. 2008;56(11):2118–23. |
|  |  |
| 56. | Bean JF, Kiely DK, LaRose S, Alian J, Frontera WR. Is stair climb power a clinically relevant measure of leg power impairments in at-risk older adults? Arch Phys Med Rehabil. 2007;88(5):604–9. |
|  |  |
| 57. | ATS Committee on Proficiency Standards for Clinical Pulmonary Function Laboratories. ATS statement: guidelines for the six-minute walk test: Guidelines for the six-minute walk test. Am J Respir Crit Care Med. 2002;166(1):111–7. |
|  |  |
| 58. | Plisky PJ, Gorman PP, Butler RJ, Kiesel KB, Underwood FB, Elkins B. The reliability of an instrumented device for measuring components of the star excursion balance test. N Am J Sports Phys Ther. 2009;4(2):92–9. |
|  |  |
| 59. | Lord SR, Fitzpatrick RC. Choice stepping reaction time: a composite measure of falls risk in older people. J Gerontol A Biol Sci Med Sci. 2001;56(10):M627-32. |
|  |  |
| 60. | Lefebvre G, Tremblay S, Théoret H. Probing the effects of mild traumatic brain injury with transcranial magnetic stimulation of the primary motor cortex. Brain Inj. 2015;29(9):1032–43. |
|  |  |
| 61. | Tremblay S, Vernet M, Bashir S, Pascual-Leone A, Théoret H. Theta burst stimulation to characterize changes in brain plasticity following mild traumatic brain injury: A proof-of-principle study. Restor Neurol Neurosci. 2015;33(5):611–20. |
|  |  |
| 62. | Kim JH, Sher S, Wang F, Berkstresser B, Shoop JL, Galante A, et al. Impact of American-style football participation on vascular function. Am J Cardiol. 2015;115(2):262–7. |
|  |  |
| 63. | Lin J, Wang F, Weiner RB, DeLuca JR, Wasfy MM, Berkstresser B, et al. Blood pressure and LV remodeling among American-style football players. JACC Cardiovasc Imaging. 2016;9(12):1367–76. |
|  |  |
| 64. | NIOSHTIC-2 Publications Search - 20022075 - Health hazard evaluation report: HETA-88-085, National Football League players mortality study [Internet]. Cdc.gov. [cited 2021 Sep 10]. Available from: https://www.cdc.gov/niosh/nioshtic-2/20022075.html |
|  |  |
| 65. | Miyake A, Friedman NP, Emerson MJ, Witzki AH, Howerter A, Wager TD. The unity and diversity of executive functions and their contributions to complex “Frontal Lobe” tasks: a latent variable analysis. Cogn Psychol. 2000;41(1):49–100. |
|  |  |
| 66. | Nissen MJ, Bullemer P. Attentional requirements of learning: Evidence from performance measures. Cogn Psychol. 1987;19(1):1–32. |
