# Supplementary material for: Multi-modality human phenotyping to examine subjective and objective health afflictions in former professional American-style football players: The In-Person Assessment (IPA) protocol
Source: PLoS One. 2022 Mar 31;17(3):e0265737. doi: 10.1371/journal.pone.0265737 (PMC8970522; doi:10.1371/journal.pone.0265737)
Supplement: S2 Appendix — (DOCX) [file pone.0265737.s002.docx]

**S2 Appendix. Participant Satisfaction Survey**.

**Enrollment**

- 1. **1.** How did you first learn about the research study?
  2. a. I got an email about it
  3. b. A fellow player suggested I join the study
  4. c. Someone telephoned me and invited me to participate
  5. d. I learned about the study from the FPHS website
  6. e. Other ______________
  7. **2.** How much time was there between being screened to actually participating in the study?
  8. a. A few weeks
  9. b. A few months
  10. c. Other ________
  11. **3.** What challenges, if any, did you encounter during the enrollment period?
  12. a. _____________________________________
  13. **4.** Do you have any recommendations for how we can continue to recruit and enroll former players?
  14. a. _______________________________________

**Study Visit**

- 1. **1.** Transportation to Boston, MA was well organized
  2. a. Strongly agree
  3. b. Agree
  4. c. Neutral
  5. d. Disagree
  6. e. Strongly Disagree
  7. **2.** Which hotel did you stay at during the length of your visit
  8. a. Wyndham
  9. b. Boxer
  10. c. Whitney
  11. d. Liberty
  12. **3.** Did you enjoy the accommodation that was provided for you
  13. a. Yes
  14. b. No
  15. c. If No, please explain why _______
  16. **4.** I received clear instructions and reminders about the days assessments throughout the study

a. Strongly agree

- 1. b. Agree
  2. c. Neutral

d. Disagree

- 1. e. Strongly Disagree

**5.** My expectation for the study matched my experience:

**6.** Overall, I was satisfied with my study experience:

**Study Staff (including technicians, physicians, clinicians, researchers)**

- 1. **1.** Staff members were friendly and explained their role within the study
  2. a. Strongly agree
  3. b. Agree
  4. c. Neutral
  5. d. Disagree
  6. e. Strongly Disagree
  7. **2.** Staff members explained the study procedures to me
  8. a. Strongly agree
  9. b. Agree
  10. c. Neutral
  11. d. Disagree
  12. e. Strongly Disagree
  13. **3.** Reviewing the informed consent form prepared me for what to expect during the study (including risks and benefits)
  14. a. Strongly agree
  15. b. Agree
  16. c. Neutral
  17. d. Disagree
  18. e. Strongly Disagree
  19. **4.** Staff members answered my questions fully and were well prepared for my visit
  20. a. Strongly agree
  21. b. Agree
  22. c. Neutral
  23. d. Disagree
  24. e. Strongly Disagree
  25. **5.** I trusted the Staff members to protect my privacy and treat me with respect.

a. Strongly agree

- 1. b. Agree
  2. c. Neutral
  3. d. Disagree
  4. e. Strongly Disagree

**6.** I was satisfied with the care I received, and felt that all my concerns were addressed.

**Results Return**

- 1. **1.** Did you feel as though the Study Physician and Medical Navigation Nurse provided you with adequate information regarding your available study results? a. Yes
  2. b. No
  3. c. If no, please explain why_______________
  4. **2.** Are you more likely to initiated lifestyle changes based off the information you received from the Exit Interview? a. Yes
  5. b. No
  6. **3.** Was the Medical navigation nurse available to answer any of my/my family’s questions and assist with getting in contact with my Primary Care Physician or referral to a specialist if needed?
  7. a. Yes
  8. b. no; if no, please explain _____________

**Future Participation**

- 1. **1.** Would you be interested in being contacted to partake in other studies with FPHS?
  2. a. Yes
  3. b. No
  4. **2.** Would you encourage other former players to participate in this study?

a. Yes

- 1. b. No
  2. **3.** Please select your reasons for volunteering for this study (selected all that apply)

a. To contribute important information to medical science

- 1. b. To potentially help other players
  2. c. To gain insights into my own health
  3. d. Because of the financial incentives of the study
  4. e. To benefit from the additional medical attention and testing that the study provided
  5. f. Other __________________________________________
  6. **4.** Have you completed or been screened for other studies with FPHS?

a. Brain Health

- 1. b. Sleep
  2. c. Goal Directed Resilience Training (GRIT)
  3. d. Personal Network
  4. e. Other ______________

**5.** Have you completed our second Health and Wellness Questionnaire?

a. Yes

- 1. b. No
  2. **6.** If you answered “no” to question 4, please select your reason for not completing the survey
  3. a. I never received it
  4. b. I would have preferred a paper survey
  5. c. It was too long
  6. **7.** If you answered “no” to question 4, would you like to receive the survey to complete?

a. Yes

- 1. b. No

**Comments**

- 1. **1.** Was there anything that could have been done differently to improve your study experience? a. ________________________________
  2. **2.** I felt that the staff members who guided me through the study visit were knowledgeable, friendly, and overall helped make the study a positive experience. f.
  3. a. Strongly agree
  4. b. Agree
  5. c. Neutral
  6. d. Disagree
  7. e. Strongly Disagree
  8. **3.** Is there anything else you would like to comment on?
